# Individualized Physiotherapy Improves Quality of Life and Symptom Burden in Advanced Cancer: A Quasi‐Experimental Study

**DOI:** 10.1002/pri.70280

**Published:** 2026-07-10

**Authors:** Gonçalo Soares, Paulo Reis‐Pina

**Affiliations:** ^1^ Department of Physical Medicine and Rehabilitation Francisco Gentil Portuguese Institute for Oncology of Lisbon Lisbon Portugal; ^2^ Faculty of Medicine Center for Palliative Medicine University of Lisbon Lisbon Portugal; ^3^ Acute Palliative Care Inpatient Unit Santa Maria Local Health Unit Lisbon Portugal

**Keywords:** neoplasms, palliative care, physical therapy modalities, quality of life

## Abstract

**Background and Purpose:**

Patients with advanced cancer frequently experience high symptom burden and reduced quality of life. Physiotherapy may represent an important nonpharmacological component of palliative care; however, evidence regarding its effectiveness in hospitalized patients remains limited. This study evaluated the effects of individualized physiotherapy on quality of life and symptom burden in hospitalized patients with advanced cancer receiving palliative care.

**Methods:**

A quasi‐experimental study with pre–post evaluation and nonrandomized allocation was conducted. Participants were hospitalized adults with stage IV cancer and a Palliative Performance Scale score of 40%–50%. Participants were allocated to an experimental group receiving physiotherapy or to a control group receiving usual care. The intervention consisted of 12 physiotherapy sessions delivered over approximately 2 weeks. Quality of life and symptom burden were assessed at the baseline and post‐intervention using the EORTC QLQ‐C30, Version 3. Mixed analysis of variance was used to evaluate changes over time between groups. Ethical approval was obtained, and all participants provided written informed consent.

**Results:**

Sixty patients were included (30 per group). Global health status and quality of life improved significantly in the experimental group, whereas the control group showed no meaningful change. Significant improvements were observed in physical, emotional, and occupational functioning. Reductions were noted in fatigue, pain, dyspnea, insomnia, nausea and vomiting, and appetite loss. Social functioning did not change significantly in either group. Most participants reported favorable perceptions of physiotherapy, with 96.7% indicating perceived benefit.

**Discussion:**

Individualized physiotherapy was associated with clinically meaningful improvements in quality of life, functional outcomes, and symptom burden in hospitalized patients with advanced cancer. These findings support the integration of physiotherapy within interdisciplinary palliative care teams. However, interpretation should consider the nonrandomized design and the short follow‐up period, which may limit causal inference and long‐term generalizability.

**Abbreviations**
CG: Control Group
EG: Experimental Group
EORTC QLQ‐C30: European Organization for Research and Treatment of Cancer, Quality of Life Questionnaire, Core 30
PC: Palliative Care
PPS: Palliative Performance Scale
QOL: Quality of Life
SB: Symptom Burden

## 1 Introduction

### 1.1 Background

Life expectancy has increased substantially in most developed countries; however, the number of years lived with illness and disability has also grown (GBD 2021 Diseases and Injuries Collaborators 2024). Cancer is a major contributor to the global disease burden, and both incidence and mortality are projected to increase in the coming decades (GBD 2023 Cancer Collaborators 2025).

In patients with advanced cancer, symptom burden (SB) is typically multifactorial and includes pain, dyspnea, fatigue, nausea and vomiting, anorexia, constipation, anxiety, and depression (Vogt et al. 2021; Newcomb et al. 2020). These symptoms may result not only from tumor progression itself, but also from anticancer treatments, functional decline, comorbidities, systemic inflammatory processes, nutritional and metabolic disturbances, and psychosocial distress (Ellis et al. 2024; Erdoğan et al. 2021).

Moreover, symptoms frequently coexist in interconnected clusters, in which physical and psychological symptoms may exacerbate one another and contribute to cumulative suffering (Gilbertson‐White et al. 2019; Amano et al. 2022). Consequently, quality of life (QOL) has become a central outcome in oncology care, providing a broader and more patient‐centered perspective than traditional endpoints such as survival or treatment response (Ng and Ozdemir 2023; Liu et al. 2025; Yan et al. 2024).

Palliative care (PC) addresses these complex needs through a proactive and holistic approach focused on relieving suffering and supporting patients and families facing life‐threatening illness (Radbruch et al. 2020). According to the World Health Organization, PC aims to improve QOL through early identification and comprehensive management of physical, psychosocial, and spiritual problems (World Health Organization 2020). Evidence consistently shows that timely integration of PC can significantly improve QOL in patients with advanced cancer (World Health Organization 2020; Gautama et al. 2023; Haroen et al. 2025).

PC is inherently interdisciplinary, involving collaboration among physicians, nurses, psychologists, social workers, and rehabilitation professionals (Radbruch et al. 2020). Within this framework, physiotherapy plays an important role in addressing functional decline and SB. Physiotherapy interventions may help maintain mobility and independence, reduce pain and fatigue, improve respiratory function, and support participation in activities of daily living (Sanders et al. 2024). In advanced illness, rehabilitation may also contribute to preserving autonomy, dignity, and social participation, while the therapeutic interaction between the physiotherapist and the patient may support emotional well‐being (Vradelis et al. 2025; Wu et al. 2025). Emerging evidence also suggests that rehabilitation interventions may support cognitive functioning through physical activity, engagement in structured activities, and promotion of attention and executive function (Vásquez‐Carrasco et al. 2025).

Consistent with this multidimensional approach, clinical guidelines from the American Society of Clinical Oncology recommend integrating interdisciplinary PC interventions aimed at improving symptom control and QOL alongside active cancer treatment (Sanders et al. 2024).

Physiotherapy may therefore represent a valuable non‐pharmacological strategy to alleviate SB and support QOL in advanced cancer; however, access to rehabilitation interventions remains limited due to resource constraints, fragmented service organization, and attitudinal barriers, including misconceptions among healthcare professionals and patients regarding the appropriateness and potential benefits of rehabilitation in advanced disease (Bernabeu‐Wittel et al. 2023; World Health Organization 2023).

Despite growing recognition of the role of rehabilitation within PC, evidence regarding the impact of physiotherapy on QOL and SB in hospitalized patients with advanced cancer remains limited. Previous studies have predominantly examined rehabilitation interventions in outpatient settings, mixed cancer populations, or broader multidisciplinary programs, with limited emphasis on individualized physiotherapy for hospitalized patients with advanced stage IV cancer receiving PC. Consequently, evidence specifically addressing this clinical context remains scarce (Hall et al. 2019; Manson et al. 2025; Gauchez et al. 2024). Evaluating the contribution of physiotherapy in this context is therefore clinically relevant and insufficiently explored.

### 1.2 Objectives

The aim of this study was to evaluate whether physiotherapy contributes to improving QOL and reducing SB in hospitalized patients with advanced stage IV cancer receiving PC.

## 2 Methods

This study is reported in accordance with the Transparent Reporting of Evaluations with Non‐randomized Designs (TREND) statement (Des Jarlais et al. 2004).

### 2.1 Clinical Trial Number

Not applicable. This study used a quasi‐experimental, non‐randomized design conducted within routine clinical care and was reviewed by the institutional Ethics Committee as a non‐clinical trial study. Participants were not prospectively assigned to intervention groups according to a research protocol; therefore, trial registration was not required.

### 2.2 Participants

Participants were hospitalized adults with advanced stage IV solid cancer and a Palliative Performance Scale (PPS) score of 40%–50% (European Portuguese version) (Sereno et al. 2022). Patients with PPS scores of 40%–50% typically require considerable assistance with self‐care activities, present reduced mobility, spend substantial periods seated or in bed, and exhibit significant functional limitations while still retaining limited participation in daily activities.

Patients presenting uncontrolled symptoms lasting more than 48 hours—such as severe uncontrolled pain, acute dyspnea at rest, persistent nausea or vomiting, severe fatigue, or other signs of acute clinical instability—were excluded to ensure patient safety and sufficient clinical stability for participation in physiotherapy. This criterion reflects routine clinical practice in the study setting, where stabilization of acute symptoms is prioritized before initiation of rehabilitation interventions.

Eligible patients received verbal and written study information and provided written informed consent. Potential participants were identified by their treating oncologist as part of routine clinical care and referred for evaluation by a specialist in Physical Medicine and Rehabilitation. After confirmation of eligibility, participants were assessed by the physiotherapist and initiated physiotherapy when clinically appropriate. Recruitment followed a non‐randomized convenience approach reflecting routine clinical conditions.

No structured physiotherapy or home exercise program was routinely offered to control participants because rehabilitation interventions were not part of standard care for hospitalized patients in the study setting. Control participants continued to receive usual oncological, nursing, and PC interventions, including pharmacological symptom management and supportive care as clinically indicated.

All assessments, interventions, and outcome measurements were conducted in the Medical Oncology ward of a tertiary public cancer center in Portugal during hospitalization.

### 2.3 Interventions

Participants were allocated to an experimental group (EG), which received physiotherapy, or to a control group (CG), which received usual medical and nursing care without physiotherapy during the study period (waiting‐list control). Participants were allocated to groups based on clinical and organizational feasibility within routine hospital care.

The EG received individualized physiotherapy based on oncological rehabilitation principles within a PC approach. Interventions targeted problems identified during clinical assessment, including reduced muscle strength, limited joint mobility, fatigue, pain, dyspnea, and limitations in activities of daily living, with the aim of improving QOL. Table S1 summarizes the physiotherapy interventions according to therapeutic aim and provides detailed information regarding dosage, frequency, and intensity parameters. All intervention parameters were individualized according to patient tolerance and clinical status and were consistent with recommendations from the European Association for Palliative Care (Payne et al. 2022), and the Multinational Association for Supportive Care in Cancer (Hart et al. 2022).

Physiotherapy was delivered through individualized face‐to‐face sessions conducted by the same physiotherapist, who had experience in oncology physiotherapy, and advanced training in PC. The intervention consisted of 12 sessions delivered over approximately 2 weeks during hospitalization. Each session lasted 20–30 min and was adapted to the patient's clinical condition and tolerance. Adherence was supported through individualized adjustment of session intensity, therapeutic communication, patient education, and involvement of family members when appropriate.

All participants continued to receive standard oncological and palliative care throughout hospitalization, including pharmacological symptom management and supportive interventions as clinically indicated. Standard palliative care was delivered according to the same institutional protocols for both groups. Acute medical conditions and symptom exacerbations were managed according to institutional protocols and were not influenced by study participation. Both groups were managed within the same clinical setting, reducing variability in routine care.

### 2.4 Outcomes

The primary outcome was QOL assessed using the European Portuguese validation of the European Organization for Research and Treatment of Cancer, Quality of Life Questionnaire, Core 30, version 3 (EORTC QLQ‐C30) (Pais‐Ribeiro et al. 2008; European Organisation for Research and Treatment of Cancer n.d.). The instrument includes functional scales, symptom scales, and a global QOL scale. Higher scores on functional and global scales indicate better functioning and QOL, whereas higher symptom scores indicate greater SB (Fayers et al. 2001). Acknowledging more recent refinements in minimal important differences (Musoro et al. 2023), clinically meaningful change was interpreted using the classic Osoba criteria (5–10 small, 10–20 moderate, > 20 large) (Osoba et al. 1998).

Secondary outcomes included functional domains (physical, emotional, cognitive, social, and occupational functioning) and SB, specifically fatigue, nausea and vomiting, pain, dyspnea, insomnia, appetite loss, constipation, and diarrhea.

Outcomes were assessed at baseline (pre‐intervention) and post‐intervention using the EORTC QLQ‐C30 under standardized conditions.

Standardized procedures were applied across participants, and the same researcher administered assessments at both time points.

Participants' perceptions of the intervention were assessed using a study‐developed 5‐point Likert scale (strongly agree to strongly disagree), pre‐tested in five patients for clarity and used descriptively.

### 2.5 Sample Size

Sample size was calculated using G*Power software for repeated‐measures ANOVA with within–between interaction (power = 0.90; effect size *f* = 0.25) (Faul et al. 2009). Because no previous studies with sufficiently comparable populations and interventions were identified, a medium effect size (*f* = 0.25), based on Cohen's conventions, was assumed for sample size estimation. The minimum required sample was 46 participants. Sixty eligible patients were included and equally allocated (30 per group).

### 2.6 Assignment Method

The unit of assignment was the individual participant. Allocation to the EG (physiotherapy) or CG (waiting‐list) followed a non‐randomized procedure based on clinical and organizational feasibility within routine hospital care. Baseline comparability between groups was assessed using appropriate inferential statistical tests. Standardized eligibility criteria, consistent intervention delivery, and uniform outcome assessment procedures were applied to reduce potential bias. Stratification or block randomization procedures were not implemented because recruitment occurred continuously within a real‐world inpatient oncology setting and depended on factors such as participant availability, clinical stability, hospital workflow, and physiotherapy scheduling capacity. More complex allocation procedures were considered impractical within the organizational constraints of routine clinical care and could have interfered with timely intervention delivery.

### 2.7 Blinding

Blinding of participants and intervention providers was not feasible due to the nature of the intervention. The study therefore followed an open‐label design.

Outcome assessment was conducted using standardized self‐reported instruments, which partially mitigated potential bias despite the absence of assessor blinding.

### 2.8 Unit of Analysis

The individual participant was the unit of analysis. Because the unit of analysis corresponded to the unit of assignment, no clustering adjustments were required.

### 2.9 Statistical Methods

Descriptive statistics summarized the characteristics of the sample. Baseline equivalence between EG and CG was assessed using Pearson's χ^2^ or Fisher's exact test for categorical variables, independent samples *t*‐tests for continuous variables, and Mann–Whitney U tests for ordinal variables. Prior to inferential analyses, normality was assessed through examination of skewness and kurtosis values. Variables with skewness < 3 and kurtosis < 7 were considered to present acceptable normality or only minor deviations from normality, consistent with Kline's recommendations. Variables violating this assumption were analyzed using appropriate non‐parametric tests. Intervention effects over time were analyzed using mixed ANOVA with one within‐subject factor (time: pre vs. post) and one between‐subject factor (EG vs. CG).

Effect size was estimated using partial eta squared (*η* ^2^ *p*), interpreted as 0.01 (small), 0.06 (medium), and ≥ 0.14 (large).

Because the constipation domain violated normality assumptions, non‐parametric tests were used. Within‐group changes were assessed using the Wilcoxon signed‐rank test (reported as *Z* values), and between‐group comparisons were assessed using the Mann–Whitney *U* test (reported as U values).

No subgroup or multivariable analyses were performed due to sample size and study design.

No missing data were observed. Statistical significance was set at *p* < 0.05 (two‐tailed).

All analyses were performed using SPSS version 29.0 (IBM Corp., Armonk, NY, USA).

## 3 Results

### 3.1 Participant Flow

**FIGURE 1:**
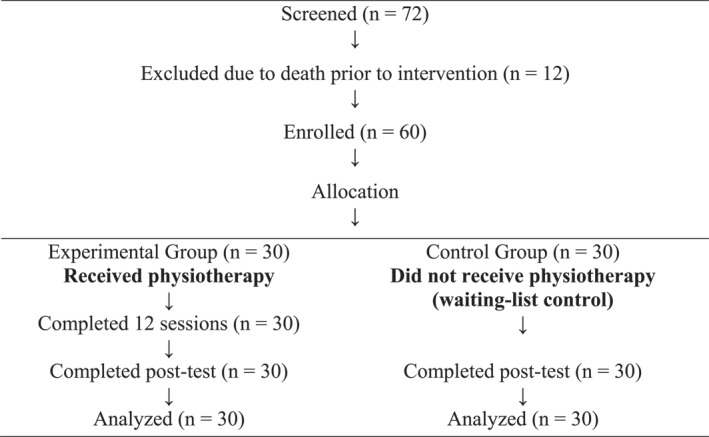
Participant flow.

Participant flow is summarized in Figure 1. No protocol deviations or losses to follow‐up occurred. Exclusions occurred only prior to intervention due to death (*n* = 12).

### 3.2 Recruitment

Participant recruitment occurred between January 2, 2024, and May 2, 2024; follow‐up was completed on June 30, 2024.

### 3.3 Baseline Data

Participants were predominantly female in both groups (EG 56.7%; CG 66.7%). Age ranged from 55 to 79 years in the EG and 55–75 years in the CG. Mean age (SD) was similar between groups: 65.83 (7.01) years in the EG and 66.53 (7.70) years in the CG (Table 1). Most participants were married or cohabiting (EG 60.0%; CG 56.7%), had secondary education (EG 50.0%; CG 56.7%), and were retired (EG 53.3%; CG 60.0%). The most common primary cancers were breast and gastrointestinal tumors (EG 50.0%; CG 60.0%). Treatment distribution was identical across groups, with most participants receiving chemotherapy (56.7%).

**TABLE 1:** Characteristics of the experimental and control groups.

Variables	Categories	Experimental group (*n* = 30)		Control group (*n* = 30)		Inferential statistics	
		*n*	%	*n*	%	Test statistic	*p* value
Sex	Female	17	56.7	20	66.7	X^2^ = 0.635	0.426
	Male	13	43.3	10	33.3		
Age range (years)	55–59	8	26.7	10	33.3	*t* = −0.368	0.714
	60–64	4	13.3	0	0.0		
	65–69	8	26.7	6	20.0		
	70–74	6	20.0	9	30.0		
	75–79	4	13.3	5	16.7		
Marital status	Married/cohabiting	18	60.0	17	56.7	Fisher = 0.952	0.954
	Widowed	6	20.0	5	16.7		
	Divorced	4	13.3	5	16.7		
	Single	2	6.7	3	10.0		
Education level	Primary	8	26.7	9	30.0	U = 413.50	0.576
	Secondary	15	50.0	17	56.7		
	Higher	7	23.3	4	13.3		
Occupation/employment status	Retired	16	53.3	18	60.0	X^2^ = 0.518	0.829
	Other	8	26.7	8	26.7		
	Employed	6	20.0	4	13.3		
Primary tumor	Breast	8	26.7	9	30.0	Fisher = 3.538	0.999
	Gastrointestinal	7	23.3	9	30.0		
	Genitourinary	5	16.7	5	16.7		
	Head and neck	4	13.3	3	10.0		
	Sarcoma	4	13.3	2	6.7		
	Melanoma	2	6.7	2	6.7		
Cancer therapy	Chemotherapy	17	56.7	17	56.7	X^2^ = 0.000	1.000
	Chemotherapy and radiotherapy	13	43.3	13	43.3		

### 3.4 Baseline Equivalence

Inferential testing showed no statistically significant baseline differences between EG and CG across sociodemographic and clinical variables, supporting group comparability (Table 1).

### 3.5 Numbers Analyzed

All enrolled participants (*n* = 60; EG = 30; CG = 30) were included in the analyses of primary and secondary outcomes and were analyzed according to assigned group.

### 3.6 Outcomes and Estimation

#### 3.6.1 Primary Outcome: Global Health Status/Quality of Life

Mixed ANOVA demonstrated a significant improvement in global health status/QOL in the EG, whereas the CG showed no significant change (Table 2). Groups did not differ at baseline but differed significantly at post‐test, with a significant group × time interaction indicating a differential trajectory favoring the intervention.

**TABLE 2:** Effect of physiotherapy on global health status/quality of life (EORTC QLQ‐C30).

Group	Pre‐test mean	Pre‐test SD	Post‐test mean	Post‐test SD	*F* value	*p* value	*η*²*p*
Experimental group	33.02	10.62	44.14	8.25	92.591	< 0.001	0.761
Control group	35.25	10.88	33.02	8.32	2.222	0.147	0.071

Effect	*F* value	*p* value	*η*²*p*
Baseline between‐group	0.641	0.426	0.011
Post‐test between‐group	27.02	< 0.001	0.318
Group × time interaction	49.949	< 0.001	0.463

*Note:* Higher scores indicate better global health status/quality of life.

Abbreviations: *η*²*p*, partial eta squared; EORTC QLQ‐C30, European Organisation for Research and Treatment of Cancer Quality of Life Questionnaire Core 30; SD, standard deviation.

#### 3.6.2 Function‐Related Outcomes

Function‐related outcomes are presented in Table 3. Overall, the EG showed improvement across several functional domains, whereas the CG remained stable or showed minimal change.

**TABLE 3:** Effect of physiotherapy on function‐related scales (EORTC QLQ‐C30).

Function	Group	Pre mean	Pre SD	Post mean	Post SD	*F* value	*p* value	*η*²*p*	Mean difference
Physical Function	Experimental	14.19	14.29	29.15	11.68	57.077	< 0.001	0.663	14.96
	Control	16.64	13.31	13.73	10.63	2.091	0.159	0.067	−2.91
Occupational Function	Experimental	23.31	19.86	30.52	15.22	6.420	0.017	0.181	7.21
	Control	28.30	19.64	26.08	19.41	0.796	0.380	0.027	−2.22
Emotional Function	Experimental	51.63	18.62	68.31	16.44	45.860	< 0.001	0.613	16.68
	Control	49.68	20.12	44.96	20.73	3.705	0.064	0.113	−4.72
Cognitive Function	Experimental	75.53	23.88	79.42	22.19	4.173	0.050	0.126	3.89
	Control	62.74	25.40	62.74	26.15	0.000	0.999	0.000	0.00
Social Function	Experimental	23.87	20.84	28.29	17.59	2.055	0.162	0.066	4.42
	Control	21.62	20.12	23.31	23.39	0.334	0.568	0.011	1.69

Function	Effect	*F* value	*p* value	*η*²*p*
Physical function	Group × Time interaction	40.078	< 0.001	0.409
Occupational function	Group × Time interaction	6.221	0.015	0.097
Emotional function	Group × Time interaction	37.880	< 0.001	0.395
Cognitive function	Group × Time interaction	1.422	0.238	0.024
Social function	Group × Time interaction	0.416	0.521	0.007

Function	Effect	Pre test			Post test		
		*F* value	*p* value	*η*²*p*	*F* value	*p* value	*η*²*p*
Physical function	Between‐group analysis	0.475	0.494	0.008	28.610	< 0.001	0.330
Occupational function	Between‐group analysis	0.959	0.332	0.016	0.974	0.328	0.017
Emotional function	Between‐group analysis	0.151	0.699	0.003	23.390	< 0.001	0.287
Cognitive function	Between‐group analysis	4.033	0.049	0.049	7.089	0.010	0.109
Social function	Between‐group analysis	0.180	0.673	0.003	0.870	0.355	0.015

*Note:* Higher scores indicate better functioning.

Abbreviations: *η*²*p*, partial eta squared; EORTC QLQ‐C30, European Organisation for Research and Treatment of Cancer Quality of Life Questionnaire Core 30; SD, standard deviation.

##### 3.6.2.1 Physical Function

Improved significantly in the EG but not in the CG. Although baseline values were comparable, the EG demonstrated significantly better physical functioning at post‐test, with a significant group × time interaction indicating a differential improvement.

##### 3.6.2.2 Occupational Function

Improved significantly in the EG but remained stable in the CG. Although between‐group differences were not significant at either time point, the significant interaction effect indicated a more favorable trajectory in the EG.

##### 3.6.2.3 Emotional Function

Improved markedly in the EG, whereas the CG showed no improvement and a slight tendency toward decline. Groups were comparable at baseline but differed significantly at post‐test, with a significant interaction effect favoring the intervention.

##### 3.6.2.4 Cognitive Function

Showed a small but statistically significant improvement in the EG, while the CG remained stable. Although between‐group differences were present at both time points, the interaction effect was not significant, suggesting similar rates of change over time.

##### 3.6.2.5 Social Function

Social function did not change significantly in either group, with no between‐group differences or interaction effects observed.

#### 3.6.3 Symptom‐Related Outcomes

Symptom‐related outcomes are summarized in Table 4, with constipation presented in Table 5. Overall, the EG showed reductions in several key symptoms, whereas the CG remained stable or showed slight worsening.

**TABLE 4:** Effect of physiotherapy on symptom‐related scales (EORTC QLQ‐C30).

Symptom	Group	Pre mean	Pre SD	Post mean	Post SD	*F* value	*p* value	*η*²*p*	Mean difference
Fatigue	Experimental	77.35	16.38	51.06	13.23	118.720	< 0.001	0.804	−26.29
	Control	65.13	19.29	70.31	18.53	2.380	0.134	0.076	5.18
Nausea/vomiting	Experimental	17.76	20.95	9.98	14.23	7.440	0.011	0.204	−7.78
	Control	24.42	24.25	23.86	27.90	0.021	0.887	0.001	−0.56
Pain	Experimental	52.75	24.79	28.31	16.46	37.790	< 0.001	0.566	−24.44
	Control	55.51	22.88	49.97	30.94	1.017	0.322	0.034	‐5.54
Dyspnea	Experimental	29.98	31.97	15.54	24.33	22.176	< 0.001	0.433	−14.44
	Control	26.64	29.53	29.98	29.48	0.816	0.374	0.027	3.34
Insomnia	Experimental	46.64	35.66	34.41	26.93	7.820	0.009	0.212	−12.23
	Control	33.31	33.89	36.63	28.14	0.281	0.600	0.010	3.32
Loss of Appetite	Experimental	54.41	34.44	45.52	29.65	6.276	0.018	0.178	−8.89
	Control	52.19	35.76	55.53	36.44	0.302	0.587	0.010	3.34
Diarrhea	Experimental	12.21	26.94	8.88	21.30	1.300	0.264	0.043	−3.33
	Control	12.21	23.93	6.66	18.34	5.800	0.023	0.167	−5.55

Symptom	Effect	*F* value	*p* value	*η*²*p*
Fatigue	Group × time interaction	57.970	< 0.001	0.500
Nausea/vomiting	Group × time interaction	2.260	0.138	0.037
Pain	Group × time interaction	7.760	0.007	0.118
Dyspnea	Group × time interaction	13.720	< 0.001	0.191
Insomnia	Group × time interaction	4.150	0.046	0.067
Loss of appetite	Group × time interaction	3.030	0.087	0.050
Diarrhea	Group × time interaction	0.355	0.553	0.006

Symptom	Effect	Pre test			Post test		
		*F* value	*p* value	*η*²*p*	*F* value	*p* value	*η*²*p*
Fatigue	Between‐group analysis	6.991	0.011	0.108	21.450	< 0.001	0.270
Nausea/vomiting	Between‐group analysis	1.296	0.260	0.022	5.893	0.018	0.092
Pain	Between‐group analysis	0.202	0.655	0.003	11.460	0.001	0.165
Dyspnea	Between‐group analysis	0.176	0.676	0.003	4.278	0.043	0.069
Insomnia	Between‐group analysis	2.201	0.143	0.037	0.098	0.756	0.002
Loss of appetite	Between‐group analysis	0.060	0.808	0.001	1.362	0.248	0.023
Diarrhea	Between‐group analysis	0.000	1.000	0.000	0.187	0.667	0.003

*Note:* Higher mean scores indicate greater symptom burden.

Abbreviations: *η*²*p*, partial eta squared; EORTC QLQ‐C30, European Organisation for Research and Treatment of Cancer Quality of Life Questionnaire Core 30; SD, standard deviation.

**TABLE 5:** Effect of physiotherapy on constipation (EORTC QLQ‐C30).

Symptom	Group	Pre Mean	Pre SD	Post Mean	Post SD	*Z* value	*p* value	Mean difference
Constipation	Experimental	37.63	35.09	18.50	25.64	−2.620	0.009	−19.13
	Control	31.45	17.90	22.20	27.57	−1.786	0.074	−9.25

Between‐group analyses		
Time point	*U* value	*p* value
Pre‐test	404.50	0.482
Post‐test	410.00	0.516

*Note:* Values are presented as 5% trimmed means. Higher scores indicate greater constipation burden.

Abbreviations: EORTC QLQ‐C30, European Organisation for Research and Treatment of Cancer Quality of Life Questionnaire Core 30; SD, standard deviation.

##### 3.6.3.1 Pain

Pain improved substantially in the EG, with no significant change in the CG. Post‐test comparisons and the significant interaction effect indicated a differential improvement associated with the intervention.

##### 3.6.3.2 Dyspnea

Decreased significantly in the EG but remained unchanged in the CG, with a significant interaction effect confirming a different trajectory between groups.

##### 3.6.3.3 Fatigue

Decreased markedly and clinically meaningfully in the EG, while the CG showed a tendency toward increased fatigue. Groups were comparable at baseline but differed significantly at post‐test, with a strong group × time interaction favoring the intervention.

##### 3.6.3.4 Nausea and Vomiting

Decreased significantly in the EG, whereas the CG remained stable. Although the interaction effect was not significant, post‐test comparisons favored the EG.

##### 3.6.3.5 Insomnia

Improved significantly in the EG, whereas the CG showed no meaningful change. Although post‐test differences were not significant, the interaction effect indicated a more favorable evolution in the EG.

##### 3.6.3.6 Loss of Appetite

Showed clinically relevant improvement in the EG, whereas the CG showed slight worsening. Although the interaction effect did not reach statistical significance, the overall pattern favored the intervention.

##### 3.6.3.7 Diarrhea

Improved in both groups, reaching statistical and clinical significance only in the CG. No between‐group differences or interaction effects were observed.

##### 3.6.3.8 Constipation

Outcomes are presented in Table 5 (non‐parametric analysis). The EG showed a statistically and clinically meaningful reduction over time, whereas the CG did not show any significant change. Between‐group differences were not significant. Because non‐parametric tests were used, interaction effects could not be formally tested; however, the overall pattern suggested a more favorable trajectory in the EG.

### 3.7 Acceptability of the Intervention (Participant‐Reported)

Most participants in the EG reported a positive perceived impact of physiotherapy on QOL: 56.7% strongly agreed and 40.0% agreed that physiotherapy improved their QOL. No participants disagreed, and only 3.3% reported a neutral position.

### 3.8 Null and Negative Findings

No significant improvement was observed in social function. Diarrhea improved significantly only in the CG. Some domains showed statistically significant but not clinically meaningful changes (cognitive function).

### 3.9 Ancillary Analyses

No subgroup or adjusted analyses were performed. Non‐parametric methods were used for constipation due to violation of normality assumptions.

### 3.10 Adverse Events/Unintended Effects

No adverse events related to the physiotherapy intervention were reported.

## 4 Discussion

### 4.1 Interpretation of Findings

This quasi‐experimental study evaluated the effect of physiotherapy on QOL and SB in hospitalized patients with advanced stage IV cancer. Physiotherapy was associated with significant and clinically meaningful improvements in global QOL in the EG, whereas the CG showed stability or slight deterioration. The significant group × time interaction indicates that these improvements were related to the intervention rather than to temporal variation.

Improvements were particularly evident in physical, emotional, and occupational functioning, as well as in key symptoms including fatigue, pain, dyspnea, and insomnia—domains that represent major contributors to suffering in advanced cancer. In contrast, cognitive and social functioning did not show consistent between‐group differences, suggesting that some domains may be less responsive to short‐term physiotherapy interventions.

Interpretation should consider potential bias related to the non‐randomized design. Although baseline comparability and the pre–post design helped control interindividual variability, residual confounding cannot be excluded. Outcomes were primarily self‐reported, which may introduce expectancy effects. Nevertheless, the magnitude and consistency of improvements across several domains, together with clinically meaningful changes according to established EORTC thresholds, support the robustness of the findings.

### 4.2 Mechanisms and Possible Explanations

The observed improvements are biologically and clinically plausible. Physiotherapy targeted mechanisms commonly affected in advanced cancer, including reduced mobility, muscle weakness, deconditioning, dyspnea, and pain. Through assisted mobilization, graded exercise, respiratory therapy, and symptom‐directed strategies, physiotherapy likely improved functional capacity and symptom control.

Fatigue reduction may reflect the use of energy‐conservation strategies and graded activity aimed at counteracting physical deconditioning. Cancer‐related fatigue is multifactorial and may result from tumor burden, systemic inflammation, treatment‐related adverse effects, anemia, reduced physical activity, sleep disturbance, and psychological distress (Zdun‐Ryżewska et al. 2025; Dantzer et al. 2025). Physiotherapy may address several of these mechanisms through graded exercise, prevention of deconditioning, energy‐conservation strategies, respiratory interventions, and promotion of functional independence.

Improvements in dyspnea may be related to respiratory physiotherapy and breathing retraining. Pain relief may derive from both physical mechanisms (mobilization, massage, transcutaneous electrical nerve stimulation) and psychological factors such as increased perceived control and therapeutic engagement. Improvements in emotional functioning may similarly reflect the therapeutic interaction and patient engagement during physiotherapy sessions. Although spontaneous symptom fluctuation cannot be excluded, the absence of similar improvements in the CG supports a true intervention effect.

### 4.3 Implementation, Feasibility, and Fidelity

The intervention demonstrated high feasibility and acceptability in a hospital palliative oncology setting. All participants in the EG completed the planned sessions, and no protocol deviations or adverse events occurred, suggesting that individualized physiotherapy is safe even in patients with advanced functional limitation. Participant‐reported acceptability was high, with most patients perceiving physiotherapy as beneficial for QOL. Intervention delivery by a single experienced physiotherapist ensured fidelity and consistency.

Implementation in broader clinical contexts may be influenced by workforce availability, institutional priorities, and organizational resources dedicated to rehabilitation in advanced cancer care. These challenges are consistent with previous research describing the role and constraints of physiotherapists in PC. A nationwide German survey reported that respiratory therapy, general physiotherapy interventions, and massage are among the most frequently used therapeutic approaches in PC, and that the standard 20‐min therapy session is often insufficient to meet patients' individual needs and fluctuating clinical conditions (Vradelis et al. 2025).

### 4.4 Generalizability

The findings are most applicable to hospitalized adults with advanced stage IV cancer and moderate functional impairment (PPS 40%–50%) treated in a tertiary oncology setting. Participants were aged 55–79 years and were receiving active oncological treatment, which may limit extrapolation to other populations.

Because the intervention was implemented in a real‐world inpatient context using standardized procedures, the results may be applicable to similar hospital oncology and PC wards. However, transferability will depend on local resources, staff training, and organizational support for rehabilitation services. As the results reflect short‐term inpatient effects, the durability of benefits beyond hospitalization remains uncertain.

### 4.5 Overall Evidence

The present findings are consistent with a growing body of literature indicating that physiotherapy and rehabilitation interventions can improve QOL and reduce SB in patients with advanced oncological disease. Previous studies have reported clinically meaningful improvements in QOL following rehabilitation‐based interventions (Myrcik et al. 2021; Ibrahim et al. 2024; Toohey et al. 2023).

Improvements in physical and functional domains align with evidence showing that physiotherapy enhances mobility, functional capacity, and independence in patients with PC needs (Myrcik et al. 2021; Putt et al. 2017; Navarro‐Meléndez et al. 2023; Maddocks et al. 2025). Improvements in occupational functioning are similarly supported by literature demonstrating that rehabilitation promotes engagement in activities of daily living through improved mobility and functional performance (Olsson et al. 2018; Volberg et al. 2023; Tiberini et al. 2025; Ozeki et al. 2024). The positive effect observed in emotional functioning is also consistent with studies suggesting that rehabilitation, therapeutic interaction, and supportive communication may enhance psychological well‐being in patients with advanced disease (Bernabeu‐Wittel et al. 2023; Myrcik et al. 2021; Putt et al. 2017; Ozeki et al. 2024; McGrath et al. 2024).

The limited effects observed in cognitive and social domains have also been reported in previous research. Cognitive impairment in PC populations is often multifactorial, and although physiotherapy may include elements of cognitive stimulation, its impact is frequently modest (Escarigo et al. 2017). Similarly, social functioning in advanced oncological disease is influenced by complex psychosocial and environmental factors and may require broader interdisciplinary or community‐based interventions beyond short‐term inpatient rehabilitation (Chrościcka et al. 2024; Ramos et al. 2025; Tennison et al. 2024; Wilson et al. 2022). Available evidence suggests that different domains of functioning in oncology patients require distinct therapeutic approaches. Cognitive outcomes have been shown to benefit from targeted interventions, including cognitive stimulation and compensatory strategy training, which may improve both objective cognitive performance and functional outcomes. In contrast, social functioning, given its multidimensional nature, may require integrated psychosocial, family‐centered, and community‐based support within an interdisciplinary palliative care approach (Nakamura et al. 2024).

Regarding SB, the present results support the role of physiotherapy as a relevant non‐pharmacological component of symptom management in advanced cancer (Myrcik et al. 2021; Putt et al. 2017; Ozeki et al. 2024; McGrath et al. 2024; Nakano et al. 2020).

Pain reduction aligns with evidence supporting physiotherapy as a complementary approach to pain control in PC (Myrcik et al. 2021; Putt et al. 2017; Ramos et al. 2025; Nakano et al. 2020; França et al. 2025).

Improvements in dyspnea are also consistent with the literature supporting respiratory physiotherapy as an effective strategy for symptom relief in advanced cancer (Ramos et al. 2025; Crombeen and Lilly 2020; Stokes et al. 2025).

The reduction in fatigue observed in the EG is consistent with studies demonstrating that rehabilitation and structured physical activity can improve energy levels in patients with advanced disease (Toohey et al. 2023; Putt et al. 2017; Ramos et al. 2025; Schunk et al. 2021; Chapman et al. 2022; Pyszora et al. 2017).

Improvements in nausea and vomiting have also been described in association with supportive physiotherapeutic strategies (Nakano et al. 2020).

Improvements in insomnia are in line with studies linking physical activity and rehabilitation to improved sleep quality (Stone et al. 2023; Høeg et al. 2025), while improvements in appetite have been associated with enhanced functional status and well‐being following rehabilitation programs (Nakano et al. 2020; Vira et al. 2021).

Although diarrhea improved primarily in the CG group, this likely reflects pharmacological management rather than a rehabilitation effect. Constipation outcomes suggested a favorable trend associated with physiotherapy, consistent with evidence linking mobility and physical activation with bowel regulation (Helgesen et al. 2024; Cui et al. 2024).

Participants also reported a highly positive perception of physiotherapy, consistent with previous studies emphasizing the value attributed by patients to rehabilitation support in advanced disease (Wilson et al. 2022; Baumbach et al. 2025; Schoeb and Bürge 2012; Stevens et al. 2017). Physiotherapists in specialized PC helped patients and families to bridge the gap between their real and ideal everyday life with the aim to maximize security, autonomy, and well‐being (Olsson et al. 2018; Wilson et al. 2022; Baumbach et al. 2025).

These findings align with broader health policy perspectives. The World Health Organization emphasizes that both rehabilitation and PC are essential components of universal health coverage and should be integrated within health systems through a multiprofessional workforce (World Health Organization 2023). Integrating rehabilitation within PC is therefore considered fundamental for improving QOL, managing symptoms, and promoting independence in individuals living with advanced and incurable diseases (Gauchez et al. 2024; Wilson et al. 2022; Cui et al. 2024).

Nevertheless, current evidence remains limited by methodological heterogeneity and risk of bias. Recent reviews indicate that although physiotherapy can positively influence patient‐reported outcomes in oncology PC, higher‐quality studies are needed to strengthen the evidence base and guide clinical practice (Putt et al. 2017; Yang et al. 2020; Baladaniya and Baldania 2025).

Overall, the present findings reinforce physiotherapy as a meaningful non‐pharmacological component of interdisciplinary PC, contributing to improved QOL and reduced SB in patients with advanced cancer (Vradelis et al. 2025; Høeg et al. 2025; Yang et al. 2020; Baladaniya and Baldania 2025; McLeod and Norman 2020; Domingos et al. 2025; Habib et al. 2024).

### 4.6 Limitations

Several limitations should be considered. First, the non‐randomized design introduces the possibility of residual confounding despite baseline comparability and the use of a pre–post analytical framework. Potential selection bias may have occurred because participants were identified by their treating oncologist and referred for specialist evaluation prior to enrollment, which may have favored patients perceived as more likely to tolerate physiotherapy. In addition, allocation to study groups was based on clinical and organizational feasibility rather than randomization, which may have resulted in an unequal distribution of unmeasured confounders between groups.

Second, the absence of participant and assessor blinding increases the risk of expectancy‐related effects, including Hawthorne, placebo, social desirability, and confirmation biases, particularly given the use of self‐reported outcome measures and direct interaction with the treating physiotherapist. These factors may have contributed to the overestimation of perceived benefits.

Third, the study was conducted in a single tertiary oncology center with a relatively small sample size, which may limit statistical power and external validity. Finally, the short follow‐up period precludes conclusions regarding the long‐term sustainability of the observed benefits.

Despite these limitations, the consistency and clinical relevance of improvements observed across multiple domains support the overall validity of the findings.

## 5 Conclusion

This study demonstrates that a structured physiotherapy intervention is associated with clinically meaningful improvements in global QOL, key functional domains, and several distressing symptoms in hospitalized patients with advanced stage IV cancer. These findings support physiotherapy as a valuable nonpharmacological component of interdisciplinary PC, even in patients with significant functional limitations.

Beyond symptom relief, the high feasibility, safety, and acceptability observed indicate that individualized physiotherapy can be successfully implemented within routine hospital oncology care. Rehabilitation therefore remains relevant in advanced disease and may contribute to maintaining comfort, autonomy, and dignity during the palliative phase.

Future research should confirm these findings through larger randomized and multicenter studies, evaluate long‐term sustainability and cost‐effectiveness, and identify patient subgroups most likely to benefit. More systematic integration of physiotherapy into palliative oncology services may represent an important step toward improving patient‐centered outcomes in cancer care.

## 6 Implications for Physiotherapy Practice

Individualized physiotherapy appears feasible and safe for hospitalized patients with advanced cancer and moderate functional limitation, even in the context of complex symptom profiles typical of advanced illness. Physiotherapists working within PC teams may play a key role in improving QOL, maintaining functional capacity, and reducing SB, particularly fatigue, pain, and dyspnea, through tailored interventions that address mobility, respiratory function, and daily activity performance.

These findings support the inclusion of physiotherapists as integral members of interdisciplinary palliative oncology teams and reinforce the relevance of rehabilitation not only in earlier disease stages but also throughout advanced illness. Timely integration of physiotherapy into inpatient oncology care pathways may enhance supportive care delivery, strengthen non‐pharmacological symptom management strategies, and promote patient‐centered outcomes focused on comfort, autonomy, and participation in daily activities.

From a service and policy perspective, ensuring adequate availability of trained physiotherapists within hospital‐based PC teams may contribute to more comprehensive and coordinated care delivery.

Future research should confirm these findings through randomized studies, evaluate long‐term sustainability of outcomes, and explore optimal intervention intensity, timing, and cost‐effectiveness to support wider implementation of physiotherapy services in palliative oncology settings.

## Author Contributions

**Gonçalo Soares:** conceptualization, methodology, data curation, formal analysis, writing – original draft, writing – review and editing. **Paulo Reis‐Pina:** supervision, methodology, validation, formal analysis, writing – review and editing. Both authors approved the definitive version of the manuscript and agree to be accountable for all aspects of the work.

## Funding

The authors have nothing to report.

## Ethics Statement

This study was approved by the Ethics Committee of the Francisco Gentil Portuguese Institute for Oncology of Lisbon, Portugal (Reference UIC/1632, December 12, 2023) and by the Lisbon Academic Medical Center, Lisbon, Portugal (Approval no. 02/24, no date). The study was conducted in accordance with the principles of the Declaration of Helsinki.

## Consent

All participants received detailed information about the study and provided written informed consent prior to participation, including consent for publication of anonymized data. Participants were assigned identification codes to ensure anonymity. To ensure confidentiality, all study records were stored on a password‐protected computer accessible only to the research team.

## Conflicts of Interest

The authors declare no conflicts of interest.

## Permission to Reproduce Material From Other Sources

The authors have nothing to report.

## Supporting information


**Table S1:** Physiotherapy interventions according to therapeutic aim.

## Data Availability

The datasets generated and analyzed during this study cannot be publicly shared due to institutional policies, which restricted the external deposition of patient‐related data. Additionally, the dataset contains sensitive clinical information from a small inpatient population, and full anonymization cannot be guaranteed without compromising data integrity. For these reasons, open deposition in a public repository is not permitted.
