# Supplementary material for: Individualized Physiotherapy Improves Quality of Life and Symptom Burden in Advanced Cancer: A Quasi‐Experimental Study
Source: Physiother Res Int. 2026 Jul 10;31(3):e70280. doi: 10.1002/pri.70280 (PMC13354070; doi:10.1002/pri.70280)
Supplement: Supplementary file 1 — Table S1: Physiotherapy interventions according to therapeutic aim. [file PRI-31-e70280-s001.docx]

**Table S1 – Physiotherapy interventions according to therapeutic aim**

| **Therapeutic Aim** | **Clinical Targets** | **Physiotherapy Interventions** | **Dosage (per session)** | **Frequency** | **Intensity** |
| --- | --- | --- | --- | --- | --- |
| Pain management | Pain relief; maintenance of mobility; reduction of muscle tension | Assisted mobilization exercises; therapeutic massage; education on self‑mobilization strategies; relaxation techniques; transcutaneous electrical nerve stimulation. | -Mobilization exercises: 10–20 min/session.  -Massage: 15–20 min/session.  -TENS: 20 min/session. | -Mobilization exercises: 1×/day.  -Massage: 1×/week.  -TENS: 1–2×/day. | -Mobilization exercises: Borg scale ≤3/10; pain-free range of motion.  -Massage: light to moderate pressure.  -TENS: 80–100 Hz (conventional) or 2–4 Hz (acupuncture-like). |
| Fatigue management | Improvement of functional capacity; optimization of energy use; promotion of activity tolerance | Therapeutic exercise; graded activity training; counseling; energy‑conservation strategies. | -Exercise: 10–30 min/session.  -Energy conservation: integrated into daily routine. | -Exercise: 3–5×/week.  -Energy conservation: throughout the day. | -Exercise: Borg scale 2–4/10; initiate with bed- or chair-based exercises in deconditioned patients. |
| Dyspnea and secretion management | Reduction of breathlessness; facilitation of airway clearance; improvement of respiratory mechanics | Autogenic drainage; manually assisted coughing; active cycle of breathing techniques; positive expiratory pressure therapy; postural drainage; diaphragmatic breathing; relaxation techniques. | -Airway clearance (ACBT/drainage): 10–20 min/session.  -Postural drainage: 5–15 min/position.  -Diaphragmatic breathing/relaxation: 5–10 min. | -Airway clearance: 1×/day.  -Postural drainage: 2–4×/day (according to secretion burden).  -Diaphragmatic breathing: 3–4×/day. | -Adjusted according to respiratory tolerance and clinical status.  -Discontinue if SpO₂ <88% OR dyspnea precludes verbal communication.  -Postural drainage: use with caution in vertebral/costal metastases. |
| Constipation management | Promotion of bowel motility; reduction of abdominal discomfort; support of mobility | Promotion of mobility; encouragement of standing and walking; abdominal massage. | -Ambulation: 3-5 min/session.  -Abdominal massage: 10–15 min/session. | -Ambulation: 2×/day.  -Abdominal massage: 1×/day. | -Ambulation: assisted or transfer-based; Borg scale ≤3/10.  -Massage: clockwise circular movements; light to moderate pressure; contraindicated in abdominal distension or pain. |
| **Abbreviations:** ACBT - Active Cycle of Breathing Techniques; Borg scale - Borg perceived exertion scale (range 0–10); TENS - Transcutaneous Electrical Nerve Stimulation. | | | | | |
